# High-resolution high-speed dynamic mechanical spectroscopy of cells and other soft materials with the help of atomic force microscopy

**DOI:** 10.1038/srep12630

**Published:** 2015-07-28

**Authors:** M. Dokukin, I. Sokolov

**Affiliations:** 1 Department of Mechanical Engineering, Tufts University, Medford, MA 02155; 2 Department of Biomedical Engineering, Tufts University, Medford, MA 02155; 3 Department of Physics, Tufts University, Medford, MA 02155.

## Abstract

Dynamic mechanical spectroscopy (DMS), which allows measuring frequency-dependent viscoelastic properties, is important to study soft materials, tissues, biomaterials, polymers. However, the existing DMS techniques (nanoindentation) have limited resolution when used on soft materials, preventing them from being used to study mechanics at the nanoscale. The nanoindenters are not capable of measuring cells, nanointerfaces of composite materials. Here we present a highly accurate DMS modality, which is a combination of three different methods: quantitative nanoindentation (nanoDMA), gentle force and fast response of atomic force microscopy (AFM), and Fourier transform (FT) spectroscopy. This new spectroscopy (which we suggest to call FT-nanoDMA) is fast and sensitive enough to allow DMS imaging of nanointerfaces, single cells, while attaining about 100x improvements on polymers in both spatial (to 10–70 nm) and temporal resolution (to 0.7s/pixel) compared to the current art. Multiple frequencies are measured simultaneously. The use of 10 frequencies are demonstrated here (up to 300 Hz which is a rather relevant range for biological materials and polymers, in both ambient conditions and liquid). The method is quantitatively verified on known polymers and demonstrated on cells and polymers blends. Analysis shows that FT-nanoDMA is highly quantitative. The FT-nanoDMA spectroscopy can easily be implemented in the existing AFMs.

Knowledge of mechanical properties of nanocomposite materials, biomaterials, cells at the nanoscale is important for both fundamental and practical applications. The mechanics at that scale defines macromechanics of tissues, composite materials^1^. In biomedical area, it has been found that the Young’s (static) modulus of cells correlates with human diseases or abnormalities, including vascular and kidney diseases, cancer, malaria, cataracts, Alzheimer, complications of diabetes, cardiomyopathies^2^,^3^,^4^ and even aging^5^,^6^,^7^,^8^. Static mechanical cues of the cell nanoscale environment define the cell fate and phenotype^9^. Study of dynamical mechanical properties of cells^10^,^11^, biomaterials^12^, nanocomposites^13^, polymers^14^ will expand our knowledge base substantially.

Storage and loss moduli are the broadly used least model-dependent quantities^15^ to describe material mechanics. Low-frequency DMS (up to 300 Hz) are the most relevant to typical physiological motions of biomaterials and cells^16^. Polymer databases of the storage and loss moduli used in industry are also limited by 300 Hz. Thus, there is a strong demand for a DMS technique capable of measuring the dynamic moduli of soft materials at the nanoscale at those relevant frequencies. Existing nanoindenters^17^ are the instruments created to do such measurements^18^,^19^,^20^. However, creep (time-dependent probe-surface contact under a constant load), non-linear elastic responses, and frequently considerable adhesion preclude the existing DMS nanoindenters from making quantitative measurements at the nanoscale even on polymers. The smallest area of the probe-surface contact, and consequently, spatial resolution of nanoindenters are typically within the micron- rather than nano- scale range^21^,^22^ (see, the Supplementary materials for detail). In the case of such soft objects as biological cells, nanoindenters cannot be utilized at all.

The next problem is related to a long time of measurement. Besides the instrumental limitations, the measurement time is fundamentally restricted by the need to wait for the creep relaxation to attain a stable contact, and consequently, quantitatively accurate measurements. This results in the measurement time per surface point (pixel) of the order of several minutes. It makes impractical both to measure fast-changing processes and to map distribution of the DMS over the sample surface. There were a few attempts to use AFM for DMS measurements^19^,^20^,^23^,^24^,^25^,^26^, however, one frequency at a time. Thus, it did not improve the time of measurements, and as explained below, negatively impacted the lateral resolution. In addition, quantitative verification of those methods is still a matter of investigation^21^,^22^.

Here we present a novel DMS approach which solves the problems mentioned above. The main idea behind our method is to record the DMS for multiple frequencies at the same time, not sequentially as presently done. Although this accelerates measurements for any material, it brings a breakthrough for soft materials. The additional substantial acceleration comes from the fact that the decreased measurement time allows avoiding waiting for the creep relaxation, which is substantial for soft materials. Moreover, avoiding the creep allows keeping the area of probe-surface contact small (the area of contact increases during creep relaxation), and consequently, attaining higher lateral resolution. Here we demonstrate recording maps of quantitative mechanical parameters for cells and polymers with lateral resolution of 50–70 nm (theoretical limit is estimated to be ~10 nm) and a temporal resolution of 0.7 sec per a point of the sample surface. These values are better than the ones obtained on polymers with the state-of-the art nanoindenter (see, Table 1): 64–150x higher in lateral resolution and 200–280x faster in speed or temporal resolution. (Note that cells were not used for comparison because it cannot be imaged with nanoindenters.) Finally, measuring the dynamic mechanical parameters for all frequencies simultaneously makes it possible to obtain DMS for exactly the same surface point, which is hard to get otherwise for highly inhomogeneous materials.

Simultaneous multi-frequency measurements have previously been used to accelerate measurements in electrochemistry (time-resolved Fourier transform electrochemical impedance spectroscopy (FT-EIS)^27^,^28^), infrared^29^,^30^, NMR^31^,^32^ spectroscopy, and in the study of rheology of complex “rheokinetic” liquids (Fourier Transform Mechanical Spectroscopy)^33^,^34^,^35^,^36^,^37^,^38^,^39^. Following the naming of the aforementioned techniques, we suggest to call this new technique Fourier Transform nanoDMA or FT-nanoDMA for short. It should be noted the described method is substantially different from so-called multifrequency AFM^11^,^40^,^41^. Briefly, those multifrequency techniques are based on a different physics (multiple resonance responses of the AFM cantilever) and do not allow measuring of DMS spectra for low frequencies.

## Results and Discussion

### Experimental setup of FT-NanoDMA method

**Figure 1:**
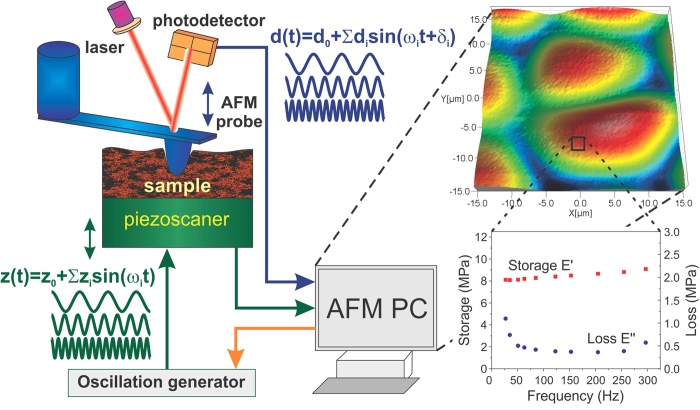
A scheme of FT-nanoDMS setup. An AFM probe indents the sample with a predefined force at each point of the mapped surface. Oscillations of multiple frequencies are simultaneously sent to a piezoscanner that oscillates the sample with amplitudes 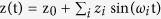 where z_0_ is the initial displacement of the sample (used to develop the sample indentation with the predefined force), *ω*_*i*_, *z*_*i*_ are the frequencies and amplitudes of each oscillation signal, respectively. The oscillations are detected through the deflection of the AFM probe 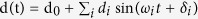, where *d*_*i*_ and *δ*_*i*_ are the amplitudes and phase shifts for each frequency, respectively (which found through the Fourier transform of the total deflection signal). The values of the recorded amplitudes and phases are used to calculate the storage and loss moduli as well as other parameters characterizing the sample.

Figure 1 shows a schematic setup of our method. At each point of the sample surface, the AFM probe indents the sample surface with a predefined vertical speed. After a predefined load force is reached, multiple harmonic oscillations are simultaneously sent to the piezoscanner that oscillates the sample with amplitudes *Z*_*i*_. These oscillations are detected as the deflection of the AFM probe *d*(*t*). Individual amplitudes *d*_*i*_ and phase shifts *δ*_*i*_ of each frequency are found through the Fourier transform of the total deflection signal *d*(*t*). The load force is defined by the need to have the indentation depth large enough to be at least 20 times higher than the deformation oscillation amplitude of the sample. It ranges from fractions of nN for the soft samples up to several μN for the hard samples.

In the described approach the forcing oscillations are applied to the sample rather than the AFM cantilever. It has as a number of advantages, for example, the decrease of influence of hydrodynamic losses due to the motion of the cantilever through medium.

The oscillations were applied to the sample for the measurement time (defined by the duration needed to resolve the amplitude and the phase of oscillations at the lowest frequency). Then, the oscillation are switched off and probe is withdrawn with a predefined speed. By analyzing the load and unload force curves, the indentation depth and static modulus were calculated. Additionally, the information about sample the creep during the oscillations was also extracted. In all our measurements, the change of the area due to creep during the oscillation was negligible (not more than 1–3%). It is worth noting that the polymer creep could cause overestimation of the elastic modulus up to ~30%^42^,^43^.

### Physics of the described implementation of FT-NanoDMA method

**Figure 2:**
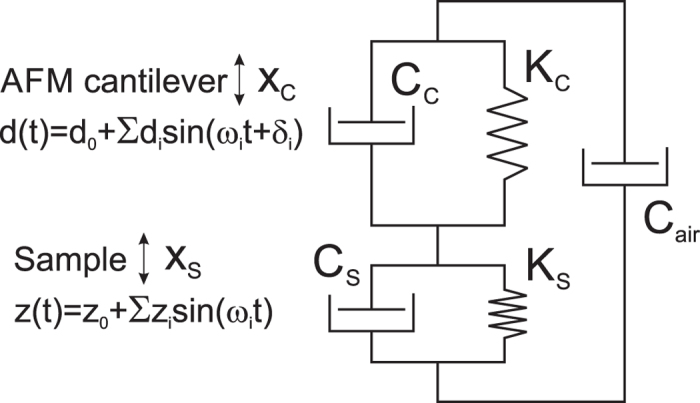
A general diagram describing the mechanical properties of all parts participating in the oscillation process. Each physical part of the system is described with an elastic spring of **K**, which obeys Hooke’s law and a viscous dashpot containing an incompressible liquid of viscosity **η**, which obeys Newton’s law.

We now describe the physics laws governing the behavior of the system. The goal is to find the storage *E*′(*ω*), loss *E*′′(*ω*) moduli, and tan *δ*(*ω*) = *E*′′/*E*′ through the measurable parameters. A general diagram describing the mechanical properties of all parts participating in the oscillation process is shown in Fig. 2. This system consists of a sample, the AFM cantilever with the attached probe, and possible coupling between those two parts though a fluid in between (can be air, water, oil, etc.). Following the standard theory of damped linked harmonic oscillators, we use both the elastic and viscous elements assigned to each part of the physical system. (Note that in contrast to the classical Maxwell and Voigt-Kelvin elastic and viscous elements, these mechanical elements are assigned to the whole physical objects rather than to a material point).

The equation describing the motion of the cantilever under the force produced by the action of the viscoelastic sample material is given by a standard formula damped harmonic oscillator under the action of an external force:
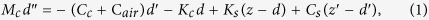
where *M*_*c*_ is the mass of the cantilever; *K*_*c*_, *K*_*S*_ on the spring constants of the cantilever and (stiffness) sample, respectively; *C*_*air*_*, C*_*s*_*, C*_*c*_ are the damping coefficients of medium (for example, air), sample and cantilever, respectively.

Here we assume no viscoelastic response of the scanner. This was independently checked by measuring cantilever response on a rigid sample (there was no visible phase shift was detected for the whole range of applied frequencies). Next simplification comes from the assumption that the frequencies of oscillation are rather far from the resonances of the system, and therefore, we can skip the retardation effects due to the finite time of propagation of the forced oscillation of the sample. Finally, we send oscillation signals *Z* = *Z*_*i*_ sin(*ω*_*i*_*t*) to the scanner. Using these assumptions one can find solutions of equation (1) as follows: instead of.
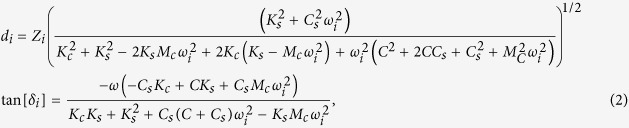
where *C* = *C*_*c*_ + C_*air*_.

The effective cantilever mass *M*_*c*_ can be estimated using the equation for a simple harmonic oscillator: *M*_*c*_ = *k*/4*π*^ 2^*f*^  2^. Here *k* is the cantilever spring constant and *f* is the cantilever resonant frequency. For example of a cantilever with k = 51 N/m and resonance frequency of 181 kHz, the effective mass is ~3.9 * 10^−11^ kg. We can now compare the “effective stiffness” originating in the inertia of the cantilever mass 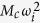 (equation 2) with the cantilever and sample stiffness K_c_ and K_s_. The maximum value of 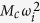 is about 1.4 * 10^−4^ N/m (at 300 Hz). This is much smaller than cantilever spring constant of 51 N/m or sample stiffness of 15–62 N/m (corneocite sample, see the below), or 7–35 N/m (pumpkin seed sample). Thus, one can ignore the terms 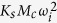 and 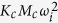 in equation (2).

An effective “damping coefficient” 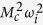 is less than 5.4 * 10^−15^ kg^2^/s^2^. This is also much smaller than the damping coefficient (for example of the pumpkin seed sample, 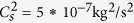). Combining this estimation with the assumption that C << Cs (which is experimentally verified by the lack of dependence of the moduli values on the oscillation amplitude, see later), one can obtain relatively simple equations
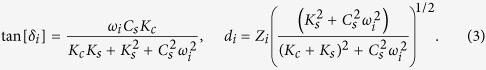
Furthermore, C_s_ *ω* << *K*_*s*_ in the majority of rigid samples. It simplifies the equations further to the following form:
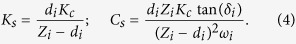
Using equations (4) and the contact mechanics equations connecting stiffness/damping and the dynamic moduli, one can find the storage *E’* and loss *E”* moduli and the phase *δ*_*i*_ for each frequency *ω*_*i*_ as follows:
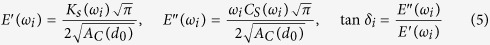
Here the contact area of the indenting probe *A*_*C*_ *(d*_*0*_) is calculated for the initial (DC) deflection of the cantilever *d*_*0*_. To find this area, the probe geometry has to be measured independently with either special calibration samples^5^,^22^,^44^ or just electron microscopy, see the Supplementary materials for more detail.

### Quantitative verifications of the FT-nanoDMA method

Particular attention was put into the development of high accuracy of the method. Multiple calibration and verifications of the method were performed. It is described in the Supplementary material in detail. Briefly, the method was first verified by using a well-characterized homogeneous PDMS polymer. Due to high chemical stability of this polymer, the values of the macroscopic storage and loss moduli (measured with independent methods, macroDMA instrument and nanoDMA mode of nanoindenter) should be equal to the ones obtained with FT-nanoDMS. Figure S2 shows the storage, loss, and static (Young’s) moduli measured on the same PDMS sample using all three techniques mentioned above. One can see an excellent agreement between all methods. A relatively high variability of the storage modulus obtained with the nanoindenter may be explained by a large range of the indenting probe which makes it sensitive to small contaminations settled on the sample surface during the measurements (it took several hours to complete).

The absence of cross-talk between frequencies was shown by simultaneous and sequential measurement of the moduli, Fig. S3. One can see an excellent agreement between the values of the moduli measured simultaneously and sequentially, in both ambient and aqueous media.

Linearity of the material (the property that is required to apply the concept of the elastic modulus) was demonstrated by measuring the response of the AFM cantilever to different oscillation amplitudes, Fig. S4. One can see no deviation from linearity in both air and water. As was demonstrated^22^,^44^,^45^,^46^, the use of an excessively sharp probe can easily result in overstretching the material which results in non-linear stress-strain response. It is typically manifest itself in a well-known phenomenon that the measured elastic modulus decreases with indentation and reaches its macroscopic value when a substantial indentation depth is reached (see^47^,^48^,^49^,^50^,^51^, and references therein). The linearity is attained here by using a sufficiently dull probe. it is important to have some way to verify that we work in the linear regime, and consequently, we can measure the moduli of elasticity. There are different ways to do that. For example, one can verify independence of the static elastic modulus of the indentation depth, see^52^. In the case of dynamic moduli, we can test the dependence, for example, of the storage modulus on the oscillation amplitude. The definitions of dynamic moduli, eq. (5) were derived based on the assumption of linear behavior of the material. Looking at the definition of the storage modulus, eq. (5) and the sample stiffness, eq. (4), one can conclude that the independence of the modulus of the oscillation amplitude can be reached only if *Z*_*i*_ − *d*_*i*_ ∼ *d*_*i*_ , or simply if *Z*_*i*_ is linearly proportional to *d*_*i*_. This can easily be verified. Two examples are shown in figure S4 which shows the results of PDMS resin tested in air and water. One can see perfect linearity within the error of measurements. The only noticeable deviation is seen for PDMS in water for the largest amplitude of 50 nm.

Next, one has to verify the lack of parasitic hydrodynamic coupling between the AFM probe and sample surface. In a viscous medium like water one should expect to see such coupling. This can contribute to viscous losses (loss tangent (tan *δ*) or the loss modulus). The lack of such contribution was tested in both water and air, Fig. S5. One can see that tan *δ* is independent of the amplitude Z up to ~15 nm, and it increases for larger amplitudes (which is consistent with contribution due to hydrodynamic coupling). To avoid this hydrodynamic coupling, hereafter we use the amplitudes smaller than 10 nm.

Finally, we did the error analysis (section 2e of the Supplementary Materials). The conclusion is that the error of the method is not higher than standard uncertainty in finding the spring constant of the AFM cantilever, i.e., ~10–20%. We also discussed a possible influence of non-ideally vertical indentation of AFM probe. This can be neglected for small indentations or at least minimize and control (section 2f of the Supplementary Materials). Thus, we can conclude that the described method can be considered as highly quantitative.

### Examples of application of FT-NanoDMA method

We now utilize the high resolution and speed of the new technique to make it possible to map dynamical mechanical properties of heterogeneous polymers and biological cells at the nanoscale. We chose the sample materials to demonstrate capability of this technique to work in the air as well in the aqueous environment with the samples of border range of the elastic modulus, from 0.5 to 1000 MPa. The maps shown below have 100 × 100 to 150 × 150 pixel digital resolution. Each pixel has DMS recorded for 10 fixed frequencies ( up to 300 Hz). While there were no limitation for the lower frequency, which we chose to be 27 Hz for demonstration purposes.

**Figure 3:**
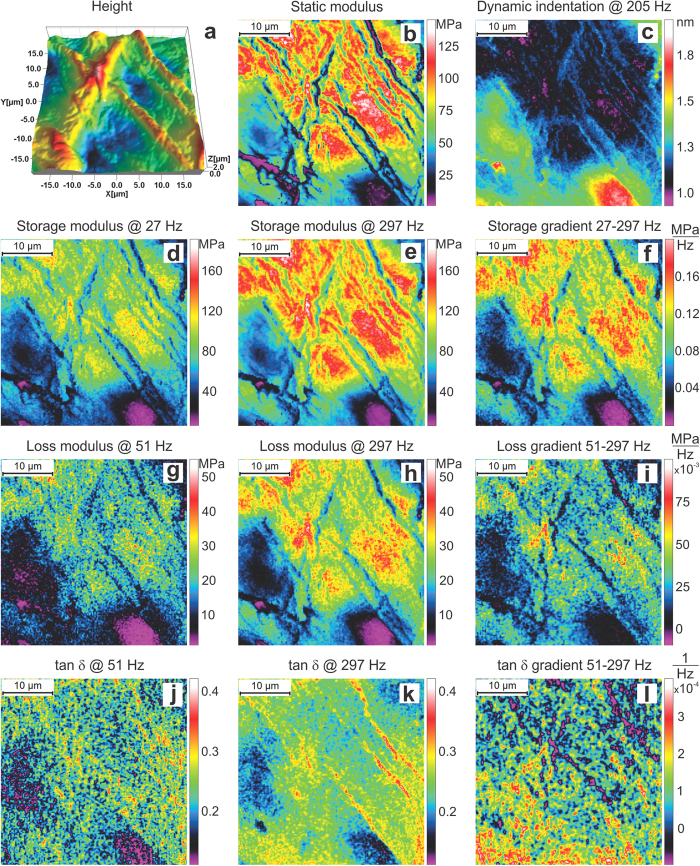
40 × 40 μm^2^ (150 × 150 pixels) dynamical mechanical maps of human corneocyte sample mapped in air at temperature of 23C. Shown are the examples of (**a**) height image, the maps of (**b**) the static elastic modulus (*E*), (**c**) dynamic indentation, (**d**,**e**) storage (*E’*), (**g**,**h**) loss moduli (*E”*) moduli, and (**j**,**k**) the loss tangent (tan *δ*(*ω*_*i*_)) measured at two different frequencies of 27 or 51 and 297 Hz; gradients of *E’, E’’*, and tan *δ*(*ω*_*i*_) calculated within these frequency range are shown in (**f**,**i**,**l**), respectively.

Figure 3 shows the maps of human corneocyte sample imaged in ambient conditions. Due to limited space, we show a small subset of a large amount of data and parameters recorded. All dynamic maps are exampled for just several frequencies (out of 10 recorded). Here we demonstrate maps of the height (a), static (b), storage (d, e) and loss moduli (g, h), loss tangent (tan *δ*) (j, k), as well as the maps of the modulus and loss tangent gradients (f, i, l). These gradients are new unique parameters that have not been reported so far, because it was impossible to measure the moduli at multiple frequencies for the same surface point at that lateral resolution. The gradients can be frequency dependent. Here we present a linearized (simplest) gradient, the slope of the moduli-frequency dependence for the frequencies within the presented range (the frequency range was chosen to show the largest changes).

One can see that the gradient maps show non-trivial variations across cells. It is possible to relate large gradients in E’’ (and tan *δ*) to particular types of molecular motion in (bio)polymers. It was shown that it could be related to main-chain and side-group motion in polymers^15^. Thus, this new gradient parameters can be a powerful tool to learn molecular organization of soft matter. From the applied point of view, one can speculate that various skin diseases and abnormalities might be identified based on such images. These maps may also be interesting in cosmetics to characterize skin mechanical properties^53^. It might practically be interesting because of simplicity of collecting corneocytes.

**Figure 4:**
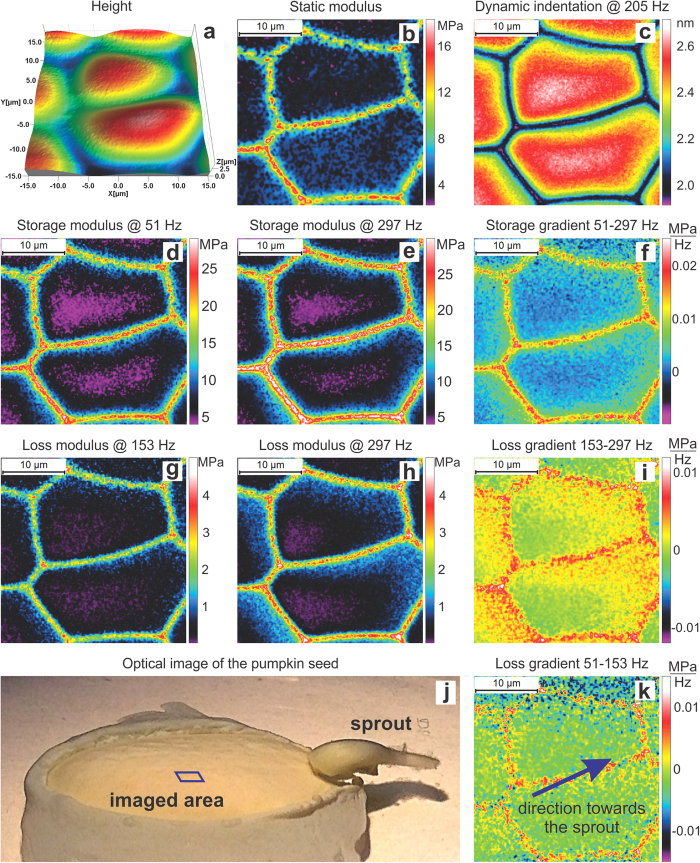
30 × 30 μm^2^ (150 × 150 pixels) dynamical mechanical maps of membrane cells of inner part of a pumpkin seed imaged in water after developing a sprout of ~1 mm in size. (**a**) Maps of height, (**b**) static modulus, (**c**) dynamic indentation, (**d,e**) storage and (**g**,**h**) loss moduli (and their frequency gradients (**f**,**i**,**k**)). The bottom row shows: (**j**) an optical image of the entire seed, the location of the imaged area, and the sprout; (**k**) loss modulus gradient and the direction towards sprout in all shown AFM images. The imaging is done in room temperature (23 °C).

Figure 4 shows mechanical maps of membrane cells of an inner part of a pumpkin seed. Maps of static (b), storage (d, e), and loss moduli (g, h) (and their frequency gradients (f, i, k)) are shown. One can clearly see softer cells and a stiffer intercellular cell wall. The cells demonstrate rather homogenous mechanical characteristics softening to the middle. One can see that the cell walls are rather heterogeneous. This seems to be plausible because nutrients should move between cells.

To demonstrate the frequency-dependent gradient of the modulus, the (linearized) gradient of the loss modulus is shown for two different frequency intervals, 153–297 Hz and 51–153 Hz. Whereas the storage modulus does not show any noticeable difference in gradient between these frequency intervals (not shown), the behavior of the loss modulus gradient is significantly different. The cells demonstrate a clear spatial directionality in the loss modulus gradient for the 153–297 Hz interval. Comparing to the optical image shown in Fig. 3, we found that the observed gradient is directed towards the sprout. Looking at other areas, we saw a similar effect. Because, no such behavior is seen in the storage map modulus, one can speculate that this visualizes a gradient of liquid nutrients moving towards the sprout. Because it is liquid, it adds to the viscous response of the cell and does change the storage (elastic modulus). As we noted previously, the gradient of the loss modulus could be linked to particular types of molecular motion in (bio)polymers^15^. Thus, the gradient seen in the loss modulus in Fig. 4 can be an indication of presence of more viscous “gel” at the cell part that is closer to the sprout (higher relative viscosity at higher frequency). Besides fundamental aspect of this knowledge, it may have some practical implementation. For example, it is plausible to expect that these gradients correlate with the speed of the sprout development. Thus, a visualization of such gradients may help to study the rate of germination and the cellular level.

### Spatial resolution of FT-NanoDMA method

To analyze the lateral resolution of the FT-nanoDMA method, we can use one of the most common definitions as the minimum distance between individual features still resolving in the AFM images^54^. It can be approximated by a formula 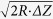, where R is the AFM probe radius, Δ*Z* is the vertical resolution of AFM. Obviously, Δ*Z* should be above noise. In our setup, we have 0.05 nm noise at 200 Hz , and R~610 nm. This implies that the lateral resolution of our setup should be >10 nm. We estimate the resolution by looking at the size of the features distinguished in the maps recorded with the FT-nanoDMA technique.

**Figure 5:**
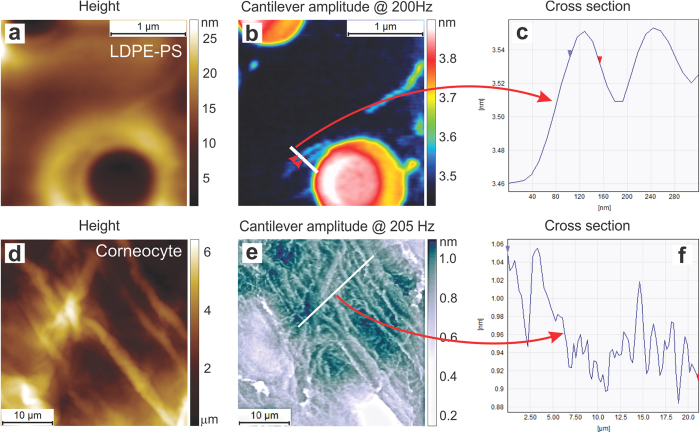
The lateral resolution of the FT-nanoDMA imaging. The cantilever amplitude maps are the basic signal used to derive the elastic moduli, see eqs (1) and (2). This amplitude is shown here because of its better visualization (it has better contrast of small features to demonstrate lateral resolution). (**a–c**) A blend of two polymers (low density polyethylene and polystyrene). (**d–f**) Corneocyte sample. (**a**,**d**) Height, (**b**,**e**) cantilev**e**r amplitude (*d*), and (**c**,**f**) the cross-sections of the cantilever amplitude are shown. The smallest features resolved in these images are of the order of 50 nm for polymers and 70 nm for the corneocytes sample.

Figure 5 demonstrates examples of FT-nanoDMA maps of a blend of two polymers (LDPE, polystyrene), and corneocytes. Height images and the cantilever amplitude *d* are shown. The cross-sections of smallest features seen in these maps demonstrate that one can attain the lateral resolution of the order of 50–70 nm. Obviously, sharper AFM probes could give a better resolution. However, the use of the excessively sharp probes can result in nonlinear stress-strain regime (see, Section 2c of the Supplementary Materials for detail).

### Temporal resolution of FT-NanoDMA method

**Figure 6:**
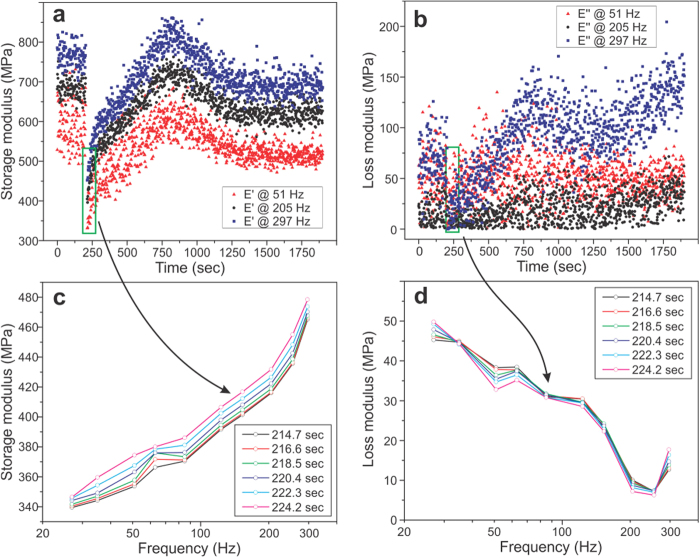
Dynamical mechanical spectra (DMS) of LPDE surface measured under fast changing temperature. (**a**) the stor**a**ge and (**b**) loss moduli for frequencies of 51, 205, and 297 Hz are shown as a function of time. A sharp drop of the modulus is the time of adding ethanol (at t = 200 second), which created near-instant heating of the medium (and consequently, the surface) by ~15 °C. Examples of DMS for (**c**) storage and (**d**) loss moduli shown with the time increment of 1.9 sec.

We now describe measurements to demonstrate temporal resolution of FT-nanoDMA. We analyze the fast kinetics of mechanical changes of a polymer (LDPE) during a fast change of temperature. Figure 6 shows an example of such fast changes of the storage and loss moduli of LDPE immersed in water, in which ethanol was introduced (to obtain 10 vol.% ethanol in water). A simple estimation shows this mixing brings up to 15 °C fast increase in the medium temperature, and consequently, softening the polymer. The changes of storage and loss moduli are shown in Fig. 6a,b, respectively. The temporal changes of three (out of 10) frequencies of 51, 205 and 297 Hz are demonstrated. Although the data can be recorded every 0.7 seconds, the time interval between spectra shown in Fig. 5 was chosen to be 1.9 seconds for better visualization.

One can see in Fig. 6a sharp drop of the modulus right after adding ethanol (at 200 seconds), and slower recovery due to cooling down the medium and equilibration of the surface temperature with the bulk of the polymer sample. A rather high heterogeneity of the modulus values could be explained not only by noise but also because each point is measured in a different (though close) location to avoid possible alteration of the surface by the AFM probe. Figure 6c, d show the storage and loss moduli as a function of frequency (dynamical mechanical spectra or DMS) measured at different points of time.

It should be stressed that in the above experiments the dynamic sample deformation was always smaller than 0.05 of the static indentation depth in our experiments. It is worth noting that the sample oscillation amplitude is not identical to the sample deformation value. For example, in the case of temperature dependence shown in Fig. 6, the static indentation depth was around 30 nm whereas the sample oscillation amplitude and dynamic indentation amplitude were 2.5 nm and 0.62 nm correspondingly. For the human corneocyte sample map (Fig. 3), the typical indentation depth, sample amplitude, and dynamic indentation amplitude were 79 nm, 2 mn and 1.2 nm, respectively.

The actual loading-unloading time for 0.7 sec force curve is 0.3 seconds (the rest 0.4 sec is the dynamic data collection time). In this case Z-piezo vertical speed is from 1 to 3 um/sec depending on the Z ramp size. The minimal vertical speed is not limited, but it could be impractical to choose it too low in many cases.

### Comparison of FT-nanoDMA and nanoindenter

To compare the spatial and temporal resolution of FT-nanoDMA and nanoindenter (the benchmark of DMS method), we studied the same sample of PDMS resin and polyurethane with these two techniques. Table 1 shows the results from the comparison (see Section 3 of the Supplementary Materials for more detail). Because it is hard to define lateral resolution for a homogeneous sample, we provide data for the diameter of contact between the probe and sample. Approximately, the lateral resolution is proportional to the contact diameter^55^. The time of measurement required to do the measurements with the nanoindenter was estimated as follows: settling of z-motor (60 sec) + settling of piezo (40 sec) + waiting for the creep relaxation (40 sec) + initial penetration (5–10 sec) + measurements of the dynamic response (1.2–3.6 sec per each frequency >30 Hz and 12 sec per frequency <30 Hz). All these times are recommended by and verified with the manufacturer.

One can see the increase of 200–280 times in the speed of measurement (consequently, temporal resolution) when using FT-nanoDMA method. It is significant. For example, it takes 1.9 hours to obtain 100 × 100 pixel image with FT-nanoDMA method. To record this amount of pixels with a nanoindenter, one would need more than 23 days, which is impractical.

The radius of contact (and consequently, the lateral resolution) definitely depends on the material of study. Higher resolution can be achieved on stiffer material. The lateral resolution of the FT-nanoDMA setup is higher than that for nanoindenter by a factor of 150x for soft materials (e.g., PDMS). A bit smaller improvement in resolution, 64–88x, were obtained on more rigid polymers (e.g., polyurethane). Note that the soft materials like biological cells, cannot be studied with nanoindenting technique at all.

In conclusion, we presented a novel method to study dynamic mechanical spectra (DMS) of soft materials (the static elastic modulus <10 GPa) with resolutions substantially better than the existing art. This allows extending the DMS measurements to the scales previously inaccessible (for example, the study of individual biological cells). The method can be used in virtually any environment suitable for the AFM operation. FT-nanoDMA is highly quantitative. Specifically, we demonstrated the linearity of the developed system (a must to use the concept of mechanical moduli), the lack of cross-talk between different frequencies, negligible contribution of hydrodynamic coupling in liquid environment when using sufficiently small amplitudes. Finally, the method was verified on several sample polymers (PDMS, polyurethane, LDPE). Maps of various mechanical properties, including the storage and loss moduli, were demonstrated on complex biological samples (corneocytes in air and cells of pumpkin seed membrane in water). New interesting information seen in the mechanical maps demonstrated the capability of the method. Being highly quantitative, we expect that FT-nanoDMA method opens a new dimension in the study of the mechanics of soft materials at the nanoscale, in particular, nanobiomaterals, and expands the knowledge base to a scale being previously inaccessible. The study of such properties will contribute to the fundamental understanding and further advances in the development of new heterogeneous polymers, nanocomposites, biomaterials, understanding cell mechanics.

## Methods

### Instrumentation, AFM mode of operation

FT-nanoDMA was built upon the base of Bruker Icon AFM. An additional piezoceramic scanner (by NPoint Inc.) was added to oscillate the sample. To record the maps of various mechanical parameters, the AFM worked in a standard indentation (single force curve) mode synchronized with the submitting and analyzing the multi-frequency signal. The data (synchronization parameters, the deflection of the cantilever and scanner vertical position) were recorded. The control of the additional scanner, synchronization with the AFM hardware was done with the help of an FPGA data acquisition and control card (by National Instruments). Scanner and cantilever oscillations were recorded with National Instruments oscilloscope card. LabView software was used to implement the control and analysis of the data. The dull AFM probes were homemade^21^ (similar probes are commercially available through various suppliers). The other standard methods used in this work for comparison are described in the supplementary materials (Sections 1). The spring constant of the cantilever was defined by using thermal tuning (built-in option in the AFM software). The other calibration methods were described in the Supplementary Materials.

### Sample preparation

Standard polydimethylsiloxane elastomer (PDMS), LDPE, and polyurethane were used (Dow Corning). A mix of polymer blends of LDPE and polystyrene was supplied by Bruker together with PeakForce QNM license. Polymer samples were mounted with epoxy on a 1cm magnetic AFM substrate (a stainless steel coin type by SPI-supplies). Ultrapure water (Millipore, Inc.) was used to study the polymers in an aqueous medium. Ethanol (ACS reagent, ≥99.5% by Sigma-Aldrich) was used for the temperature change experiments.

Corneocytes were obtained from human skin. The samples were collected with a double sticky tape and stored in Petri dish for 24 hours to equilibrate with the surrounding environment. All experimental protocols used in this work were exempt from the regular Institution Review Board (IRB) review by the IRB committee of Tufts University.

Seeds from pumpkin (*Cucurbita pepo*) were open to expose the top part covered with the seed membrane. The bottom part of the seed was fixed in a Petri dish with epoxy. Sterile water (filtered with 200 nm filter) was added. After that the seed was kept in the Petri dish under quiescent conditions in room temperature for 3 days. A sprout of 1 mm was developed by the beginning of the experiments.

### Supportive methods used in this work

#### Nanoindentation

Hysitron TI 950 TriboIndenter indentation system (Hysitron Inc., Minneapolis, MN) was used to perform indentations and record force-displacement curves on all polymer samples. A cono-spherical diamond probe with a radius of 108 μm, as provided by the manufacturer, was utilized in all experiments. A standard calibration procedure, which included the load-frame compliance, was performed before each experiment.

All samples were tested in the load-control mode. Indentations on three different regions were performed for each sample. To minimize viscoelastic response, the pattern of three segment experiment was used with loading and unloading times of 5 sec and holding at the maximum load for 10 seconds. The creep drift in each indentation experiment was ≤0.2 nm/s. All experiments were performed at room temperature (22–24C) and relative humidity of 40–60%. The elastic modulus was calculated from the unloading part of load-penetration curves using standard Oliver-Pharr method^56^,^57^.

NanoDMA mode (NanoDMA III) was used as follows. The storage and loss moduli were also measured at three different regions of each sample. The measurements were performed at 5–400 μN load with the oscillation amplitudes of 5–40 nm and the frequency ranging from 10 Hz to 300 Hz. All experiments were performed at room temperature (22–24C) and relative humidity of 40–60%.

#### DMA

The macroscopic (hereafter, bulk) rigidity moduli were determined using the TA Instruments Q800 Dynamic Mechanical Analyzer (DMA). A standard 12 mm compression clamp holder was used in all measurements. The measurements were performed under controlled force/strain rate for the static mode of operation. To determine the static bulk modulus, a simple quasistatic compression mode was used (the sample was clamped in between two parallel 12 mm round plates). The static modulus was calculated from the slope of the stress/strain curve. This study was conducted at room temperature (22–24 C) using the maximum force up to 18 N. The bulk values of the Young’s modulus were calculated using the strain less than 0.1% (as recommended by the manufacturer).

Dynamic parameters of the material, such as storage and loss moduli were measured in the multi-stress/strain mode (using the same clamp setup). In this mode, the cycling loading was applied to the sample at the rate of 10–100 Hz and the maximum drive force up to 10N N (which resulted in the amplitude of ~15–30 μm).

#### Mechanical model used to calculate the static elastic modulus using the AFM data

To calculate the static elastic modulus, the measured force-indentation curves were processed with the help of the DMT model.
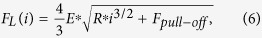
where *F*_*L*_ is the load force, E^*^ is reduced Young’s modulus: E^*^ = *E*/(1 − *v*^2^), *v* is the Poisson ratio, R^*^ is the reduced radius 1/R^*^ = 1/*R*_*indenter*_ + 1/*R*_*surface*_, and *i* is the indentation depth, *F*_*pull–off*_ is related to adhesion (more precisely, the force at the point of “pull-off” of the AFM probe, or pull-off force for short). The pull-off force can easily be found from the force indentation curves.

The maximum vertical compressive stress σ_max_ are reached at the center of the contact circle at the maximal load, *F*_max_ in the sample indented with a spherical probe (is found using the following equation^58^,^59^:
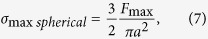
The contact diameter is an important value that can be refereed as the lateral resolution of the indentation method. The contact diameter *d* can be calculated though the following equation:
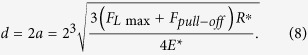


## Additional Information

**How to cite this article**: Dokukin, M. and Sokolov, I. High-resolution high-speed dynamic mechanical spectroscopy of cells and other soft materials with the help of atomic force microscopy. *Sci. Rep.* **5**, 12630; doi: 10.1038/srep12630 (2015).

## Supplementary Material

Supplementary Information

## Figures and Tables

**Table 1:** Comparison of FT-nanoDMA method with existing nanoindenter method.

**Property**	**TI 950 Hysitron nanoindenter- based nanoDMA**	**AFM-based FT-nanoDMA**
Contact diameter	22,000 or 33,000 nm (PDMS)	160 or 230 nm (PDMS)
	9,000 or 15,000 nm (polyurethane)	140 or 170 nm (polyurethane)
Minimum vertical indentation	1000 or 2600 nm (PDMS)	100 or 400 nm (PDMS)
	100 or 300 nm (polyurethane)	5 or 10 nm (polyurethane)
Total measurement time at one point of the surface	>200 sec (for 10 frequencies)	~0.7–1 sec (for 10 frequencies though the number of frequencies is not a limiting factor)
Time to record 100 × 100 pixel map	23 days (impractical)	1.9 hours
Ability to study individual biological cells	no	yes
Frequency range	Up to 300 Hz	Up to 300 Hz

The comparison is done on the same samples of two polymers: PDMS resin (the Young’s modulus of 1.5 MPa) and polyurethane (the Young’s modulus of 0.63 GPa).
