# Supplementary material for: High-resolution high-speed dynamic mechanical spectroscopy of cells and other soft materials with the help of atomic force microscopy
Source: Sci Rep. 2015 Jul 28;5:12630. doi: 10.1038/srep12630 (PMC4649865; doi:10.1038/srep12630)
Supplement: Supplementary Information [file srep12630-s1.pdf]

Supplementary materials

for

High-resolution high-speed dynamic mechanical spectroscopy of cells and other soft materials with the help of atomic force microscopy

M. Dokukin[1], and I. Sokolov[1,2,3,*]

[1] Department of Mechanical Engineering, Tufts University, Medford, MA 02155

[2] Department of Biomedical Engineering, Tufts University, Medford, MA 02155

[3] Department of Physics, Tufts University, Medford, MA 02155

1. Measurement of the AFM probe geometry

The radius and shape of the AFM probes were evaluated by imaging a standard TipCheck sample (TIP001, Aurora NanoDevices, Canada). The shape and area of the probes were reconstructed using TipCheck-sample method (built-in option of Bruker Nano, Inc AFM software). Fig.S1 shows an example of the probe of spherical shape with the radius of 670±50 nm. All probes were measured twice, after the first indent on the polymer surface with the maximal load, and after all measurements, to exclude possible probe damage.

All probes used in this work had a shape very close to a semi-sphere up to 200 nm from the apex. The geometry of the TipCheck sample doesn't allow characterizing probe at higher distances. We could assume based on SEM images (not shown) that these probes are still spherical at the distance up to 2/3 of their radius from the apex.

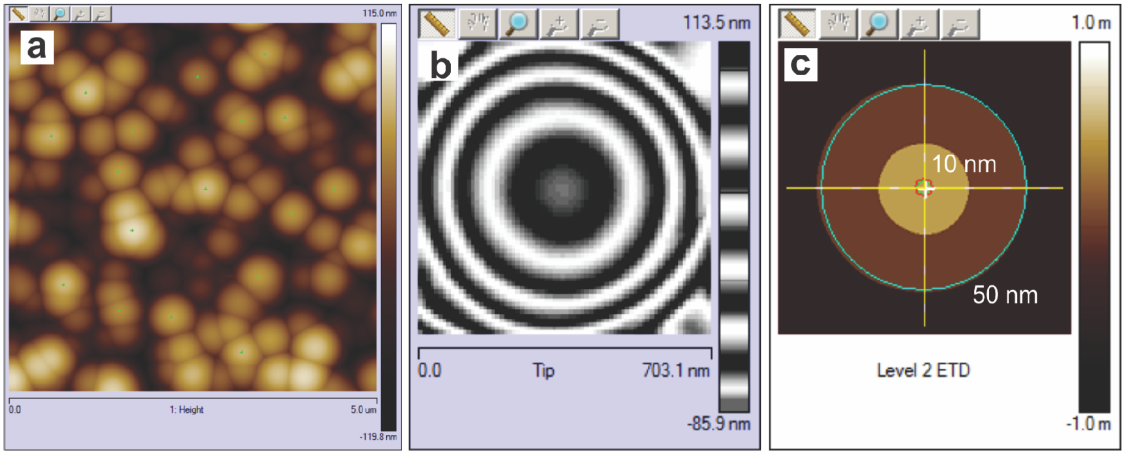

Figure S1 | Geometry of the AFM probe measured with the tip-check sample. (a) Topography of the TipCheck sample imaged with the dull R=670±50 nm probe, (b) shape of the probe reconstructed with Bruker AFM software, and (c) the probe cross-sections at 10 and 50 nm distance from the apex.

2. Verifications of the FT-nanoDMA method

a. Comparison of the moduli obtained with FT-nanoDMA method and well-established techniques

The accuracy of the method was verified on well-characterized homogeneous polymer, PDMS. Here we assumed that the values of the macroscopic storage and loss moduli (measured with independent methods, DMA instrument, nanoDMA mode of nanoindenter) should be equal to the ones obtained at the nanoscale with FT-nanoDMS. PDMS was chosen as it is one of the most homogeneous polymers known. Fig.S2 shows the storage, loss, and static (Young's) moduli measured on the same PDMS sample using all three techniques mentioned above. One can see that all moduli are indeed quite similar.

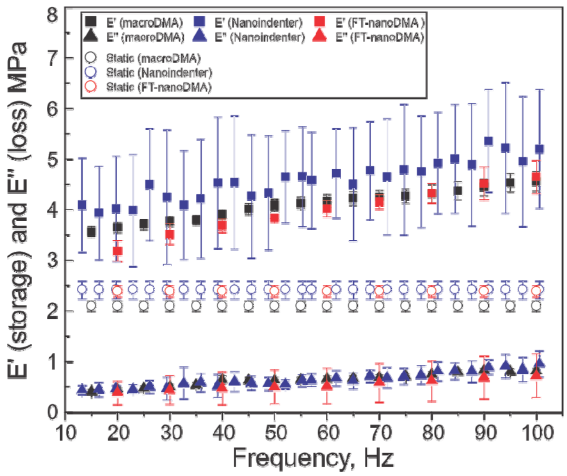

Figure S2 | The results of the comparison of the storage, loss, and static (Young's) moduli measured with macroDMA (TA Instruments Q800 DMA), nanoDMA (Hysitron nanoindenter), and the FT-nanoDMA method.

b. The absence of cross-talk between frequencies

A multi-frequency method on the base of atomic force microscopy (AFM) and FPGA electronics was implemented. We demonstrated feasibility of using 10 different frequencies simultaneously to measure the storage and loss moduli of soft polymers (PDMS, polyurethane, and polystyrene samples; static elastic modulus was in MPa –GPa range). The correct work of the implementation was verified by comparing the obtained results with those when measured using the same frequency range sequentially.

Figure S3 shows an example of such verification for a sample of PDMS resin. Each point shows the average and one standard deviation for measurements done at ten different points of the surface, and with 3 different amplitudes (the results should be amplitude-independent). Dynamic calibration of the AFM setup was done for the whole frequency range of 3-300Hz. A sinusoidal -signal of 2.5-- 10 nm amplitude was sent to the piezo-scanner in two different modes: One frequency in a time and 10 frequencies all at once. The dull probe used in this experiment had the geometry close to an ideal spherical shape with a radius of curvature of 1000± 120 nm. The cantilever and scanner positions were recorded with the sampling rate of 250kHz. One can see an excellent agreement between measurements when 10 frequencies were measured sequentially and when measured simultaneously.

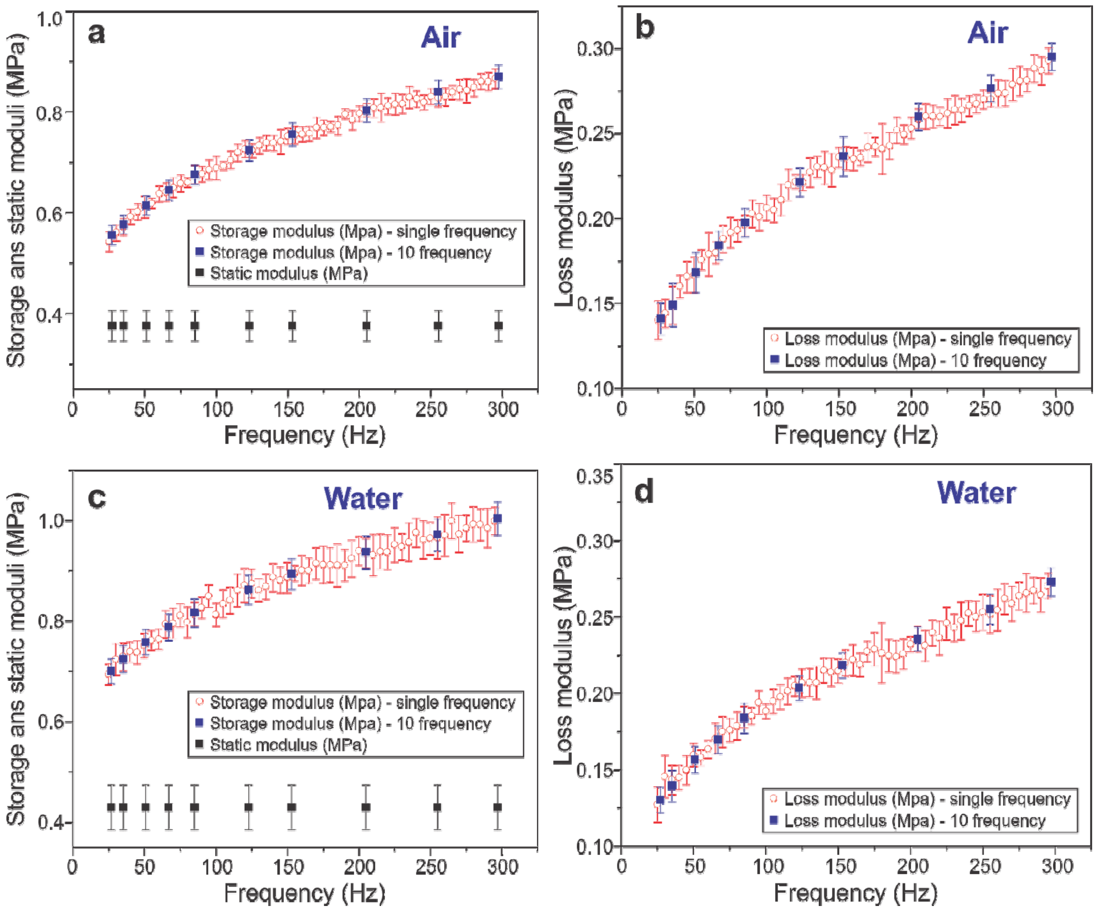

Figure S3 | Measurements of PDMS resin in air and water. A representative example of verification of the dynamic moduli measured for 10 frequencies simultaneously and one in a time. The storage (E') and loss (E'') moduli are shown.

c. *Linearity and the minimum radius of the probe*

The resolution of FT-nanoDMA is defined by the diameter of the contact between the indenting probe and sample. The diameter of contact is defined by the static load and adhesion force between the sample and probe. At first glance, such a diameter can be minimized by using a sharp probe. However, as was demonstrated [1,2], the use of an excessively sharp probe can easily result in overstretching the material (non-linear stress-strain response). To minimize the stress, one needs either to increase the area of contact between the sharper indenter and sample or to use a duller indenter. As a result, the diameter of contact for the linear stress – strain regime is about the same for both dull and sharp indenters. The optimal radius $R*$ of the indenting probe that gives the best resolution is given by the following formula [1]:

$$R^* = \frac{6w_{adh} E^{*2} + \sqrt{6}\sqrt{\pi E^{*2} F_{L\max} \sigma_{\max}^3 + 6 w_{adh}{}^2 E^{*4}}}{\pi^2 \sigma_{\max}^3}, \tag{S4}$$

where $F_{L\max}$ is the maximum load force allowed for the linear stress-strain relation, E* is the reduced Young's modulus of the material, $\sigma_{\max}$ is the maximum vertical compressive stress, w_{adh} is the *adhesion* energy (per unit area) of interaction of two flat interfaces made of the materials of the AFM probe and sample. As a matter of rule, the lateral resolution is higher on more rigid samples.

An example of full analysis of polyurethane was done in [3]. It was found that the maximum stress corresponding to the linearity limit was ~60MPa (with the help of macro DMA instrument), w_{adh}≅0.035 J/m^2. Taking the maximum load force or 50 nN, one can find that the AFM probe radius should be not less than 210 nm be able to work in the linear stress-strain regime.

The probes used in this work have radii in the range of 550-1000nm. This is a bit excessive, but gives additional guarantee that we do not reach the linearity limit. This brings another important question, how to find the linearity limit in the case of heterogeneous materials at the nanoscale, and in the case of materials which cannot be scaled up to macroscopic size (for example, biological cell). The study of this question is beyond the scope of this work; it will be studied in the future. Two examples are shown in figure S4. PDMS resin is tested in air and water. One can see perfect linearity within the error of measurements. The only noticeable deviation is seen for PDMS in water for the largest amplitude of 50nm.

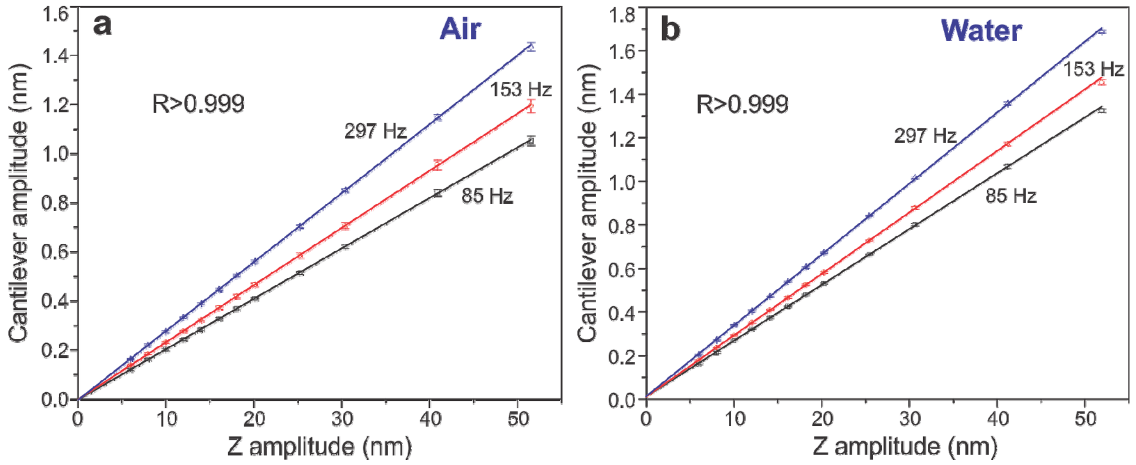

Figure S4 |Tests of linearity for PDMS resin in air and water.

d. Possible contribution of liquid medium to damping

Starting from equation (3), we neglected a possible contribution of damping of the fluid environment. There is a relatively simple way to test it. Measuring the viscous parameters the (loss modulus or tangent of loss angle (loss tangent)) for a homogeneous isotropic polymer, one should see no difference in the values of those viscous parameters when changing the oscillation amplitude Z_i (as soon as the amplitude is much less than static indentation). If such dependence is seen starting from some large amplitude, it presumably means that our approximation of equation 3 was not correct, and one has to take into account damping due to the liquid environment.

Figure S5 shows the measurements of loss tangent ($\tan\delta$) for a homogeneous and isotropic polymer (PDMS) as the function of amplitude Z_i (three different frequencies are shown). One can see that $\tan\delta$ is independent of the amplitude up to 15nm (and higher in air). Some changes in $\tan\delta$ is seen in water for higher amplitudes. These may be considered as the limit up to which we can neglect damping of the medium. It should be noted that 15 nm is quite large amplitude, and there is no any particular need to work in such regime.

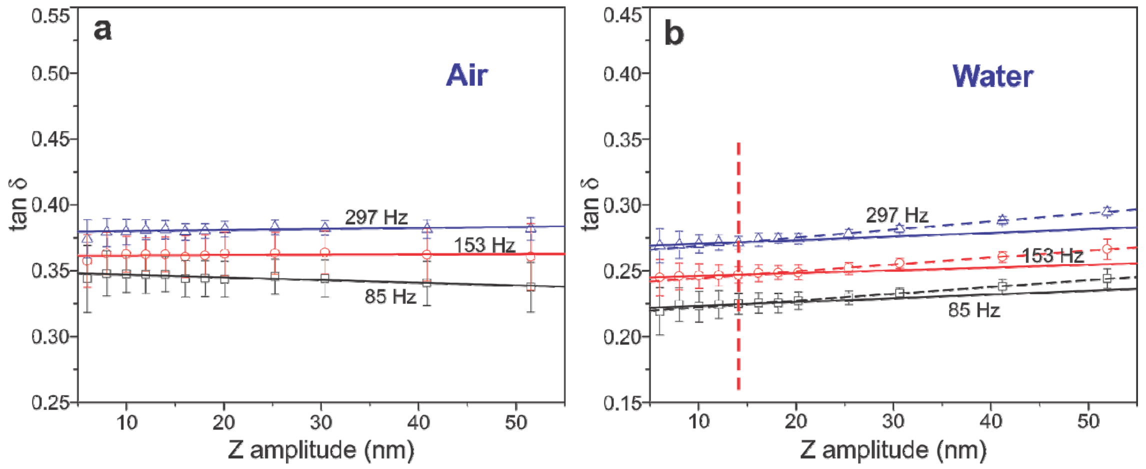

Figure S5 | Verification of the absence of noticeable damping for PDMS in air (left) and water (right). Dependency of $\tan\delta$ on the oscillation amplitudes Z_i is shown.

e. Error analysis

To exclude coincidental agreement of the rigidity moduli for the surface and bulk, we analyze possible errors in calculation of the moduli obtained in this work. The main source of the measurement errors in the AFM experiment typically originates from inaccurate determination of the size and shape of the indenter probe [4], not well-known Poisson ratio, uncertainty in the determination of the point of contact (and as a result indentation depth), the error in measuring the spring constant of the AFM cantilever, from the surface and probe roughness, scanner and sample creep, AFM photo-detector nonlinearity[5-8]. In our work we exclude AFM photo-detector nonlinearity and scanner creep by using the close-loop scanner and calibrating the photo-detector as described by the manufacturer. The roughness in the has to be taken with special models or to effectively decreased by a decrease in the initial indentation. Comparing to the static modulus measurements, the dynamic measurements described here are less dependent on roughness, because it is squeezed to some extent with the initial indentation load. We analyze the influence of the other errors on the elastic moduli calculated here.

The error in the static modulus coming from inaccuracy in definition of the probe-sample contact point was evaluated in a number of papers, see [2,3]. The error of calculating the storage and loss moduli should be substantially smaller because it comes from the error of definition of the area of probe surface contact, which is defined with less uncertainty than vertical position of the probe at the moment of the contact. For example, the radii of the dull

probes used in this work have the error of 10%. According to eq. (S8), it results in the error of ~5% in the definition of the storage and loss modulus.

As one can see from the modulus definitions, the error coming from the uncertainty of the spring constant of the AFM cantilever equals to the error of the elastic moduli. It is commonly accepted that the spring constant of the AFM cantilever can be measured with 10-20% error. This implies that we should expect 10-20% error in the definition of the rigidity modulus. It should be noted that this error, being the same multiplicative factor for all measured moduli, does not change the dependence of the storage and loss moduli on frequency.

Similar equations can be used to estimate the error due to the uncertainty in the Poisson ratio. If we take the uncertainty of the Poisson ratio, for example, between 0.4 to 0.5, the error in the calculation of the elastic modulus is ~10%. Again, being the same multiplicative factor for all measured moduli, this uncertainty does not change the dependence of the storage and loss moduli on frequency.

As to the error coming from the creep effect of the sample, it was found that the polymer creep could cause overestimation of the elastic modulus up to ~30% [4,9]. As to the FT-nanoDMA method, the creep effect can be neglected because it gives less uncertainty to the contact area then the one coming from the error in definition of the probe shape (creep can be measured directly).

f. Influence of non-ideally vertical indentation of AFM probe.

This phenomenon is a known potential problem when using the AFM probes indentation because the probes are attached to a *tilted* cantilever. This actually was one of the reasons of designing nanoindenters in which the indenting probes move vertically downward. This issue is schematically shown in Figure S6 (left). If the consequent lateral shift is excessively large, it can lead to interference with the vertical response of the material. If the lateral shift is not too large, the lateral shift is not as serious of a problem as it might look. The lateral and vertical deformations can still be considered independently while working within the proportionality elastic limit. However, if the lateral shift becomes too large, the measurements can be out of the proportionality elastic limit. Moreover, the *optical system of detection of the cantilever deflection can become nonlinear*. The former effect can be detected by searching nonlinearity as previously described. The later effect can be taken into account by not exceeding a maximum allowable deflection limit of the cantilever (such a limit is clearly seen in the calibrating plots against a rigid surface, Fig.S6 (right)). *In either*

case, it is possible to choose the appropriate AFM cantilever to minimize these effects. For example, a long (but still stiff) cantilever will decrease the described problems. Finally, when using spherical probes with indentation depths much smaller than the probe radius, it obviously decreases the described effect due to probably sliding between the probe and sample (in particular, when oscillating the probe via applied additional sinusoidal signal).

As we demonstrated, [1,2] AFM indentation can be used to derive macroscopic values of homogeneous polymers with rather high accuracy. Moreover, the modulus values were found to be independent of the indentation depth, which means a lateral shift. *These results mean that the effect of non-ideally vertical indentation of AFM probe is negligible when the appropriate cantilever and probe are used.*

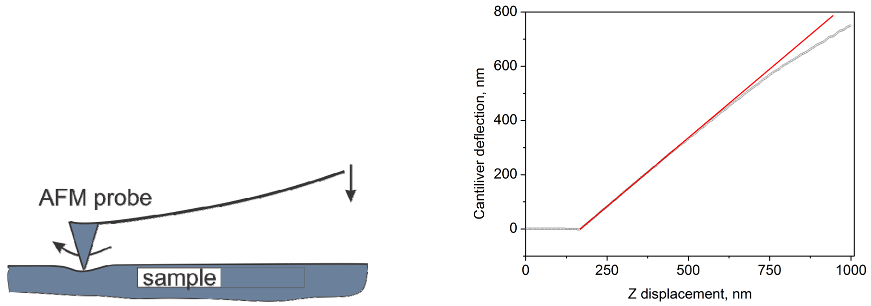

Figure 6. Left: A schematic of the AFM probe indentation. An excessively large lateral shift of the probe during indentation may interfere with the vertical response of the material.

Right: The deflection of the cantilever may become nonlinear for the excessively large load force. An example of such deviation from linearity is clearly seen in the calibration plot (cantilever deflection versus Z displacement of the scanner).

3. Comparison of FT-nanoDMA technique with existing top-of-the-line nanoindenter (Hysitron TI950)

Here we compare of the FT-nanoDMA method with the existing top-of-the-line nanoindenter (Hysitron TI950) with respect to the lateral resolution and time of measurement. We did the comparison with respect to a) the lateral resolution (minimum contact diameter of the indenters that allows for deriving stable values of the elastic moduli within the linear stress – strain regime), and b) the time of measurement of the signal comprised of a set of 10 different frequencies (ranging between 10 and 300 Hz). PDMS and polyurethane (freshly cleaved) polymer samples were tested. The comparison of FT-nanoDMA and the current nanoindenter bench-mark, the Hysitron TI950, is summarized in Table 1. The measurements using the FT-

nanoDMA method are described in the main text. The measurements done with Hysitron nanoindenter are described below.

i. Lateral resolution

The lateral resolution of the contact canning probe methods is limited by the diameter of the indentation contact. When doing indentation on PDMS resin, the nanoindenter has a contact diameter of ~20 µm when its indentation reaches *stable values* of the elastic modulus, and 33 µm when the values get close to the microscopic value (and the stress under the indenter is within the proportionality limit of the strain-stress ratio). The AFM setup described has a contact diameter of ~160 and 230 nm under similar conditions. In the case of polyurethane, these numbers are 9 or 15µm and 140 or 170nm for the nanoindenter and AFM, respectively. Thus, *the lateral resolution of the proposed AFM setup is higher than that for nanoindenter by 150x for PDMS and 64-88x for polyurethane*.

It should be mentioned that this increase in resolution is rather weekly dependent on the details of definition of the contact diameter. Specifically, we defined the lateral resolution to be the diameter of the contact when indentation reaches values of the elastic modulus close to the macroscopic value. This makes sense because of the high homogeneity of PDMS. However, some skeptics may reason that such a definition of lateral resolution is an excessively *conservative definition*. In principle, one can reach the stable values of the modulus well before approaching the macroscopic values. It is a well-known issue that the elastic modulus typically decreases with indentation and reaches its bulk value when a substantial indentation depth is reached (see, [10,11], [12,13], [14] and references therein). We demonstrated [2] that the higher values of the elastic modulus near the surface is frequently a result of nonlinearity (and sometimes because of not taking adhesion into account). Nevertheless, if we ignore those results, because they are not generally accepted as of yet, we may correlate the lateral resolution with the *diameter of the contact when indentation reaches stable values of the elastic modulus ("stable reading" definition).*

Figure S7 demonstrates these two possible definitions of the lateral resolution for the example of measuring the static elastic modulus of PDMS with a Hysitron nanoindenter and FT-nanoDMA as described above. One can see that "conservative" resolution is ~230nm FT-nanoDMA vs 33µm of Hysitron nanoindenter; whereas the "stable reading" resolution is 160nm (FT-nanoDMA) vs 22 µm (Hysitron). Thus, *the lateral resolution of the FT-nanoDMA setup is higher than that for nanoindenter by a factor of ~150x for soft materials (like PDMS) independently of definition of the lateral resolution*.

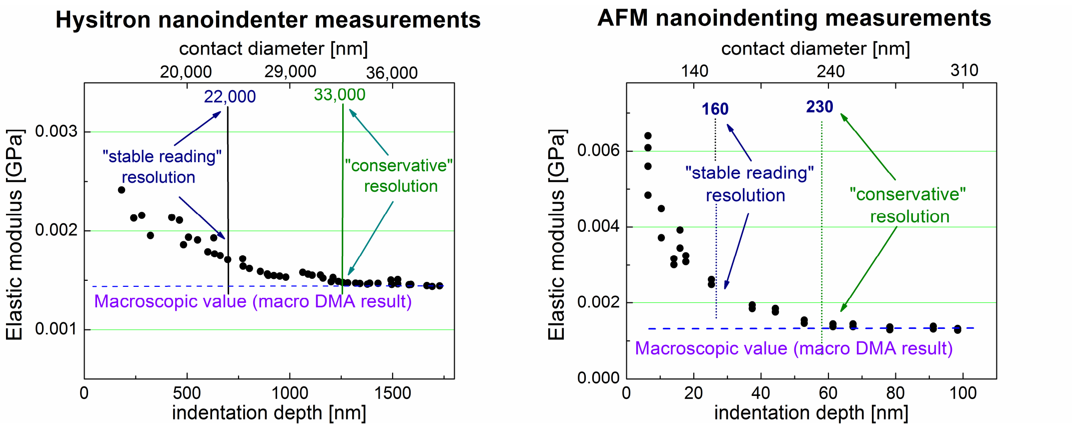

Figure S7. Different definitions of lateral resolution when measuring the elastic moduli by means of FT-nanoDMA and nanoindenter. PDMS study is shown. The microscopic modulus (measured with macroDMA) is reached only after developing a sufficient indentation. It is plausible to expect reaching the macroscopic value of the modulus at the nanoscale because of high homogeneity of PDMS resin.

<u>Note</u>: A possible confusion with recent high-resolution high-speed mechanical mapping techniques

New high-resolution high-speed rigidity mapping AFM techniques have been recently introduced by Witec (Digital Pulse mode), NT-MDT (Hybrid Mode), Veeco/Bruker (HarmoniX™ and PeakForce™) [15]. These methods allow for measuring some sort of elastic modules, say at 512x512 surface points, as fast as 30-90 min. Both techniques use a rather <u>*high*</u> operational (and <u>*single*</u>) frequency (tens and hundreds of kHz for HarmoniX and ~KHz for the other modes). Besides using just a single frequency, this frequency is too high to be interesting for soft materials. Finally, the modulus derived with those modes cannot be directly translated into the storage and loss modulus. The value of the elastic modulus in these modes is approximated by static modulus calculation (using typically the DMT model) despite the force curves are collected in a highly dynamic way. It makes this "modulus" dependent, for example, on the load force. Although this modulus can be close to the expected values of the static modulus for materials weakly dependent on the indentation speed (see, e.g., ref.[3]), these methods can be treated as relative imaging rather than truly quantitative.

References

1 Dokukin, M. E. & Sokolov, I. Quantitative Mapping of the Elastic Modulus of Soft Materials with HarmoniX and PeakForce QNM AFM Modes. *Langmuir* **28**, 16060-16071, doi:10.1021/la302706b (2012).

2 Dokukin, M. E. & Sokolov, I. On the Measurements of Rigidity Modulus of Soft Materials in Nanoindentation Experiments at Small Depth. *Macromolecules* **45**, 4277-4288, doi:Doi 10.1021/Ma202600b (2012).

3 Dokukin, M. E. & Sokolov, I. Quantitative Mapping of the Elastic Modulus of Soft Materials with HarmoniX and Peak Force QNM AFM Modes. *Langmuir* **28**, 16060-16071, doi:Doi 10.1021/La302706b (2012).

4 Moeller, G. AFM Nanoindentation of Viscoelastic Materials with Large End-Radius Probes. *J Polym Sci Pol Phys* **47**, 1573-1587, doi:10.1002/polb.21758 (2009).

5 Cappella, B. & Dietler, G. Force-distance curves by atomic force microscopy. *Surf Sci Rep* **34**, 1-103 (1999).

6 Butt, H. J., Cappella, B. & Kappl, M. Force measurements with the atomic force microscope: Technique, interpretation and applications. *Surf Sci Rep* **59**, 1-152, doi:DOI 10.1016/j.surfrep.2005.08.003 (2005).

7 Lin, D. C., Dmitriadis, E. K. & Horkay, F. Robust strategies for automated AFM force curve Analysis-II: Adhesion-influenced indentation of soft, elastic materials. *J Biomech Eng-T Asme* **129**, 904-912, doi:Doi 10.1115/1.2800826 (2007).

8 Lin, D. C., Dimitriadis, E. K. & Horkay, F. Elasticity of rubber-like materials measured by AFM nanoindentation. *Express Polym Lett* **1**, 576-584, doi:DOI 10.3144/expresspolymlett.2007.79 (2007).

9 Ngan, A. H. W. & Tang, B. Viscoelastic effects during unloading in depth-sensing indentation. *Journal of Materials Research* **17**, 2604-2610 (2002).

10 Alderighi, M., Ierardi, V., Fuso, F., Allegrini, M. & Solaro, R. Size effects in nanoindentation of hard and soft surfaces. *Nanotechnology* **20**, -, (2009).

11 Briscoe, B. J., Sebastian, K. S. & Adams, M. J. The Effect of Indenter Geometry on the Elastic Response to Indentation. *J Phys D Appl Phys* **27**, 1156-1162 (1994).

12 Belikov, S., Erina, N., Huang, L., Su, C. M., Prater, C., Magonov, S., Ginzburg, V., McIntyre, B., Lakrout, H. & Meyers, G. Parametrization of atomic force microscopy tip shape models for quantitative nanomechanical measurements. *J Vac Sci Technol B* **27**, 984-992, doi:Doi 10.1116/1.3071852 (2009).

13 VanLandingham, M. R., Villarrubia, J. S., Guthrie, W. F. & Meyers, G. F. Nanoindentation of polymers: An overview. *Macromol Symp* **167**, 15-43 (2001).

14 Clifford, C. A. & Seah, M. P. Quantification issues in the identification of nanoscale regions of homopolymers using modulus measurement via AFM nanoindentation. *Applied Surface Science* **252**, 1915-1933, doi:10.1016/j.apsusc.2005.08.090 (2005).

15 Sahin, O., Magonov, S., Su, C., Quate, C. F. & Solgaard, O. An atomic force microscope tip designed to measure time-varying nanomechanical forces. *Nat Nanotechnol* **2**, 507-514 (2007).
